# 18F-FDG Positron-Emission Tomography/Computed Tomography Findings of Radiographic Lesions Suggesting Old Healed Pulmonary Tuberculosis and High-risk Signs of Predicting Recurrence: A Retrospective Study

**DOI:** 10.1038/s41598-019-49057-5

**Published:** 2019-08-29

**Authors:** Yu Ji, Chunchun Shao, Yong Cui, Guangrui Shao, Jingsong Zheng

**Affiliations:** 1 Department of PET/CT, Shandong Cancer Hospital and Institute, Shandong First Medical University and Shandong Academy of Medical Sciences, 440 Jiyan Road, 250117 Jinan, Shandong China; 2 Department of Radiology, The Second Hospital of Shandong University, 247 Beiyuan Rd, 250033 Jinan, Shandong China; 3 Department of Evidence-Based Medicine, The Second Hospital of Shandong University, 247 Beiyuan Rd, 250033 Jinan, Shandong China

**Keywords:** Tuberculosis, Risk factors

## Abstract

Pulmonary tuberculosis (PTB) is a common worldwide infection with high mortality and morbidity, especially in developing countries. This study analyzed PET/CT findings in tumor patients with radiographic lesions suggesting old healed pulmonary tuberculosis (OHPTB) and imaging follow-up to find and verify PET/CT signs that may predict tuberculosis recurrence. A retrospective analysis of the tumor patients was carried out. These patients underwent 18F-FDG PET/CT in our center from 2010 to 2018. Confirmation of tuberculosis recurrence was obtained by follow-up of morphological changes in old lesions by PET/CT or CT. In total, 238 patients with a complete medical history were included in the final study, and 22 patients experienced OHPTB recurrence. We found that the SUVmax of tuberculosis in PET/CT was significantly increased in the recurrence group compared to the non-recurrence group [5.00 (3.40, 7.30) vs. 1.10 (0.80, 1.30), P < 0.001]. The ROC curve showed good discrimination, with an AUC of 0.980, and a cut-off SUVmax value of 2.15 was identified (the sensitivity was 90.5%, the specificity was 97.2%, the positive predictive value was 76.0%, and the negative predictive value was 99.1%). Both the tumor and the anti-tumor treatment can cause the patient to be immunocompromised and might further cause the recurrence of OHPTB. Positive imaging on 18F-FDG PET can predict the recurrence of OHPPT. Although there might be a false positive, 18F-FDG PET can greatly narrow the monitoring range. A negative result on imaging has high reliability for eliminating the possibility of tuberculosis recurrence. PET/CT has important clinical significance in tuberculosis management in patients with concurrent OHPTB.

## Introduction

Tuberculosis (TB) is a common infectious disease caused by mycobacterium tuberculosis, especially happens in the lungs. Reports showed that there are about 10.4 million new cases of TB and more than 200 million people in the incubation period in 2015^1,2^. Radiographic findings suggested that the old healed pulmonary tuberculosis sequelae lack of clinical or microbial evidence is one of the most dangerous factors for the development of active TB^3^. Although routine imaging studies can reveal morphological changes in OHPTB to determine recurrence, it is difficult to predict the risk of recurrence of OHPTB. For example, conventional CT examination can be used to accurately diagnose OHPTB; however, it can only provide morphological changes that are obviously inadequate for predicting the recurrence of tuberculosis. Molecular changes usually need much less time than morphological changes; therefore, PET imaging may be better able to facilitate the prediction of the recurrence of tuberculosis, even if the resolution is not high. The combination of PET and CT can not only accurately diagnose OHPTB at the anatomical level but also predict the risk of recurrence at the molecular level.

As a noninvasive imaging technique, 18F-FDG PET/CT is widely used in tumor imaging and can reflect the metabolism of tissue cells at the molecular level. 18F-FDG also gathered accumulates in inflammatory cells, and has been widely used in inflammatory and infectious diseases; therefore, 18F-FDG absorption can be observed in PTB and other related diseases^4,5^. However, OHPTB monitoring is a long-term process, and PET/CT examinations are expensive and cannot be routinely performed. Therefore, PET/CT findings with regard to OHPTB have only been reported in a few cases, and the PET characteristics that may predict tuberculosis recurrence have not been verified.

This institution is a specialized hospital for cancer prevention and treatment. The patients are all tumor patients, 18F-FDG PET/CT scans are routinely performed. Large number of cancer patients with OHPTB and many times of follow-up provide an opportunity for exploring the PET/CT values in old pulmonary tuberculosis recurrence. Patient immunity is impaired by both tumors and anti-tumor treatments, disrupting the balance between tuberculosis and the immune system, which can induce latent tuberculosis recurrence. This provides the perfect conditions for identifying and validating the PET/CT characteristics of OHPTB recurrence. Moreover, tumor patients need multiple PET/CT or CT examinations over a long time, meaning that this study did not add to the patient’s financial burden or radiation dose.

In this study, our aim was to identify OHPTB lesions with a potential risk of recurrence in immunocompromised patients. To achieve this goal, we selected newly diagnosed tumor patients. The tumors and anti-tumor treatments had reduced their immunity, potentially inducing the recurrence of latent tuberculosis. We searched for 18F-FDG PET/CT characteristics that predict the recurrence of pulmonary tuberculosis. This research analyzed the PET/CT findings of 238 patients with OHPTB and followed up with imaging to identify the PET/CT values that can predict the recurrence of pulmonary tuberculosis.

## Results

**Figure 1:**
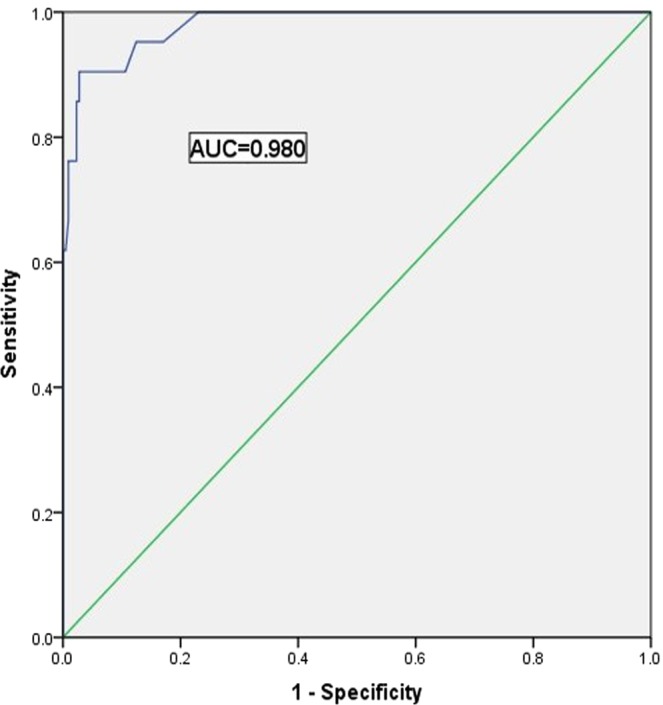
Receiver operating characteristic (ROC) curves of SUVmax. The cut-off value of SUVmax was 2.15 to distinguish recurrence patients from non-recurrence people. The area under the curve was 0.980 (95%CI 0.958 to 1.000).

In total, 964 tumor patients with radiographic lesions suggesting OHPTB were screened. More than 1 year of follow-up, 238 patients with complete medical histories were included in the final study, and 22 cases encountered tuberculosis recurrence. When compared with the non-recurrence patients, the SUVmax of lesions was significantly higher in the recurrence patients [5.00 (3.40, 7.30) vs. 1.10 (0.80, 1.30); P < 0.001]. There were no significant differences in age and sex between the recurrence and non-recurrence group. SUVmax cut off value was 2.15 for distinguishing the recurrence from the non-recurrence. According to the ROC curve, the SUVmax results were statistically significant. The area under the curve was 0.980 (95% CI 0.958 to 1.000), and 2.15 was the maximum cut-off value. At this point, the sensitivity was 90.5%, and the specificity was 97.2%. The positive predictive value was 76.0%, and the negative predictive value was 99.1% (Fig. 1).

**Figure 2:**
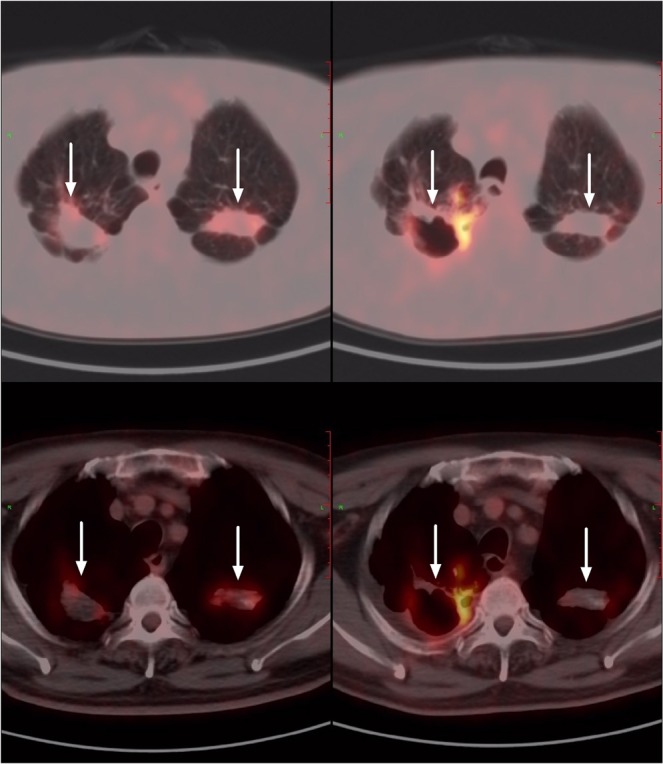
A 70-year-old man with history of colon cancer. Old tuberculosis lesions were seen at the apex of both lungs. The SUVmax = 3.1 (right) and SUVmax = 3.2 (left) on the first PET/CT scan. Six months after surgery and systemic chemotherapy, PET/CT showed that the volume of the old tuberculosis foci on the right side increased significantly. There were cavities in the lesion, new flaky dense shadows around them, and the SUVmax increased to 7.5. Interestingly, the metabolism of the left old tuberculosis decreased (SUVmax = 1.5).

**Figure 3:**
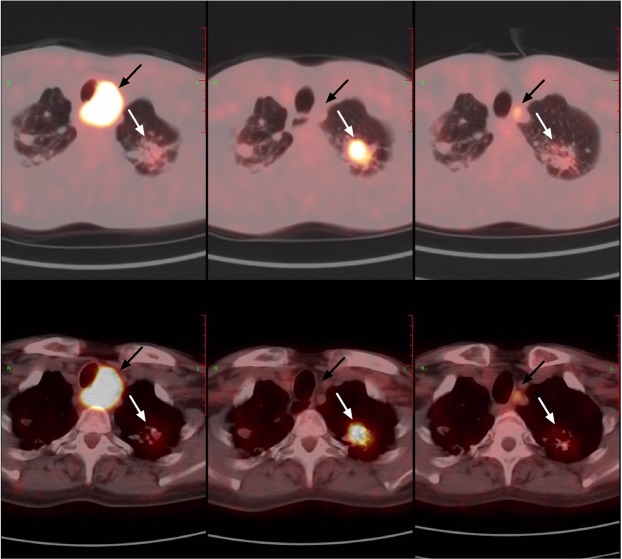
A 60-year-old man with history of lung cancer. Old tuberculosis lesions were seen at the apex of both lungs, and a metastatic lymph node can be seen beside the trachea. After 5 months of chemotherapy, the PET/CT follow-up showed that the volume of old tuberculosis in the left apex pulmonis increased, and the SUVmax increased from 2.6 to 7.5 (white arrow); the paratracheal high metabolism lymph node volume was reduced and metabolism was essentially disappeared (black arrow). After 4 months of anti-tuberculosis treatment, the PET/CT follow-up showed that the metabolism of hypermetabolic tuberculosis decreased, but the volume of metastatic lymph nodes increased slightly and metabolism increased again.

**Figure 4:**
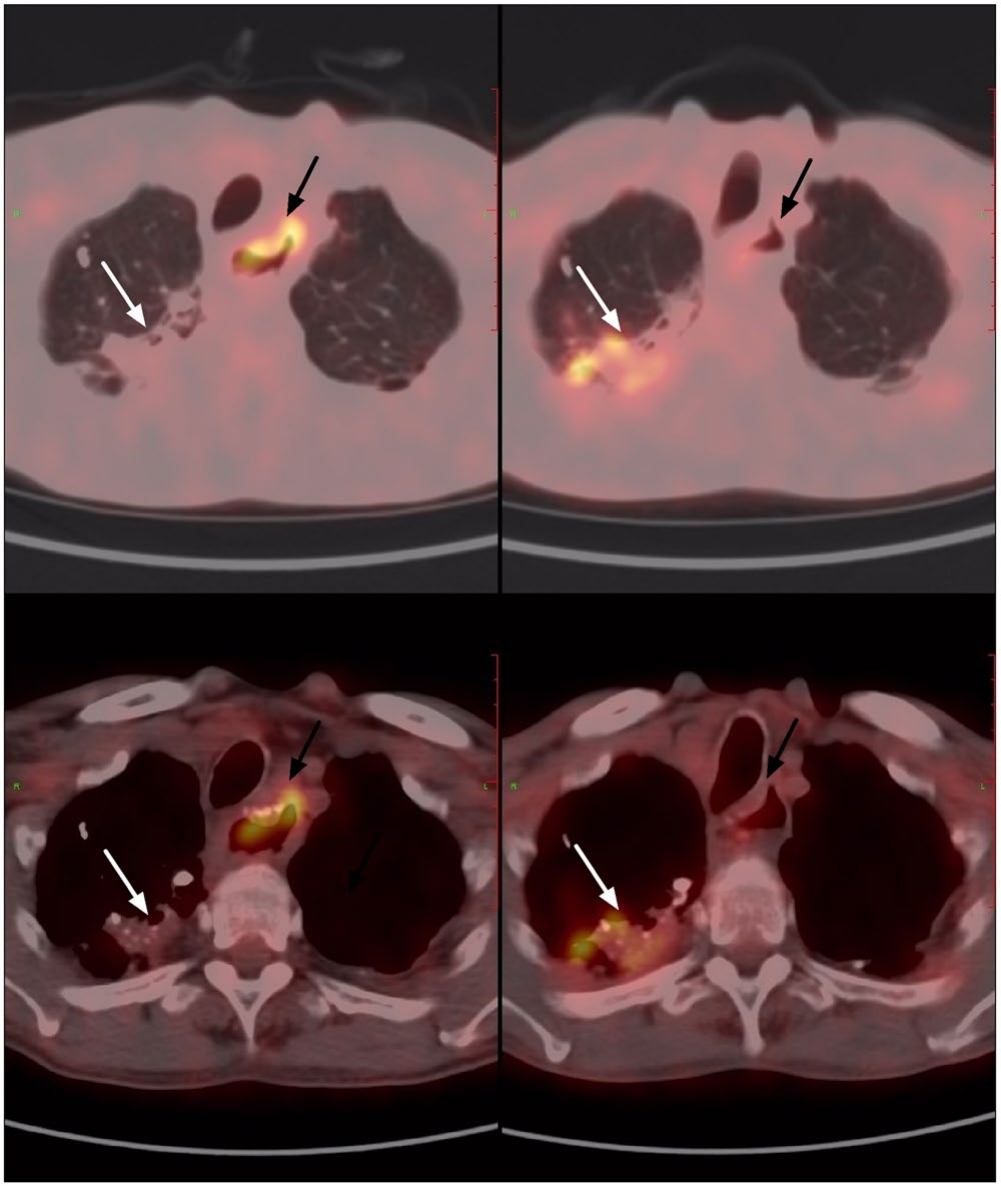
A 54-year-old man with history of esophageal cancer. Old tuberculosis lesions were seen at the apex of right lung (white arrow), and the FDG hypermetabolism anastomosis after esophageal cancer surgery (black arrow). 1 year later, the PET/CT follow-up showed that the volume of old tuberculosis increased, and the SUVmax increased from 1.6 to 4.0.

The CT results of the patients with tuberculosis recurrence were complex, as most were mixed with multiple forms of radiological recurrence characteristics. For example, there were morphological changes in the old tuberculosis lesions and new lesions with different shapes. In 22 patients with tuberculosis recurrence, only 3 showed enlargement of the primary lesion, and the other 19 showed mixed changes (Fig. 2). Twelve patients with confirmed recurrence had at least one PET/CT follow-up examination. Among them, 7 patients received anti-TB therapy: 5 patients had OHPTB lesions with reduced metabolisms and volumes (Fig. 3), and 2 patients experienced treatment failure, with increased lesion metabolism, increased lesion volume and new lesions. In the other 5 untreated cases, 3 patients had marked enlargement of the old lesions and increased lesion metabolisms (Fig. 4), 1 patient had decreased lesion metabolism and volume, and 1 patient had no change in lesion size or metabolism.

## Discussion

Pulmonary tuberculosis is a common infectious disease worldwide, and to date there is no absolute cure for it. Owing to the acceptable rates of treatment failure and disease recurrence after the discontinuation of chemotherapy, the currently standard treatment of period is 6 months^6^. Importantly, the bacteria granuloma is not always eliminated, may be dormant. What kind of outcome depends on the host’s immune status and lead to a spectrum of TB states from no infection, latent infection and subclinical disease to overt active disease^2,7^. Many studies have shown that impaired host immunity is a predisposing factor for PTB, cell-mediated immune deficiency related conditions, such as HIV infection, malignancy, malnutrition, renal disease, diabetes, and other immunosuppressive therapies^8–11^. In addition, many researchs about genotyping *M. tuberculosis* isolates with RFLP showed that the radiographic features are often similar in patients with primary tuberculosis and patients who relapse after treatment^12,13^. On the other hand, an important feature of radiologic is dependent on the host’s immune level rather than the elapsed time of infection, and the time of the acquisition infection to the development of clinical disease cannot reliably predict whether tuberculosis recurrence.

In about 5–10% of the infected population, endogenous reactivation of a latent infection develops many years after the initial infection^14,15^. The focal or patchy heterogeneous consolidation in primary lesions is the most common radiographic manifestation of reactivated PTB, which has relatively high oxygen tension and impaired lymphatic drainage^16^. Another common finding is the presence of poorly defined nodules and linear opacities (tree-in-bud sign), which are found in about 25% of cases^17^. Cavity is the other radiologic hallmark of reactivated TB, about 20–45 percent of patients are evident with this radiographically characteristics, and the necrotic material along the endobronchial spread may result in TB infection in the same or others lobes^17^. The morphological manifestations of OHPTB recurrence are different from those of primary or metastatic tumors, making the differential diagnosis relatively easy. Patients in whom the diagnosis is difficult can have the diagnosis confirmed by follow-up, and very few patients who are difficult to differentiate are excluded from this study.

A study by Malherbe *et al*. found that when PET/CT imaging was performed immediately after standard anti-tuberculosis treatment, 86% of patients had active uptake in residual pulmonary tuberculosis lesions, but the vast majority of the patients were clinically cured^18^. One year after treatment, PET/CT showed that 68% of the patients still had residual 18F-FDG-positive lesions, although the size and metabolic intensity of most residual lesions had improved, suggesting that the majority of patients still have persistent inflammation lesions^18^. Therefore, the increase in 18F-FDG uptake in tuberculosis is not a specific adverse outcome. The reason for the high positive rate of PET imaging in tuberculosis residual lesions may be due to the PET/CT scan time points. the length of a cure for PTB. MTB in the lung tissue may last a few months after culture negative has been achieved by antibiotics. Autoimmunity is an important complement to the elimination of MTB, but this process takes longer. Malherbe *et al*. performed PET/CT follow-up for a relatively short time, when MTB was still in the process of being cleared. This study also showed a trend toward a decrease and disappearance of FDG uptake in residual tuberculosis lesions over time. However, in our study, the end time of anti-tuberculosis treatment averaged more than 20 years. Therefore, the scan time point is an important reference factor for explaining the FDG uptake of residual tuberculosis lesions and requires continuous follow-up.

In this study, the SUVmax in the recurrence group was higher than that in the non-recurrence group [5.00 (3.40, 7.30) vs. 1.10 (0.80, 1.30); P < 0.001], indicating a higher risk of recurrence of hypermetabolic lesions. Although high uptake in PTB lesions may suggest active disease, it may also indicate a host immune system response that will eventually prevail. However, the immune response is more active in hypermetabolized lesions on PET. When immunosuppression occurs in the host, if the lesion contains residual *M. tuberculosis*, it is more likely to evolve into recurrent tuberculosis^1^. In this study, not all PET-positive patients experienced tuberculosis recurrence, which may be due to the absence of active M. tuberculosis in the positive lesions or the lack of disruption of the immune balance. Other research has found PTB lesion metabolic activity in the patients after a clinical cure and they did not present with recurrence on follow-up^18^. Therefore, the clinical status of the patient must be carefully linked with the evidence of metabolic activity when interpreting the findings of the OHPTB. The SUVmax of recurrent lesions was significantly different from that of non-recurrent lesions, suggesting that 18F-FDG uptake is associated with the number and virulence of tuberculosis bacteria. Some studies^19–21^ also found that when PET/CT was used to monitor the efficacy of tuberculosis treatment, the SUVmax of the lesion decreased when the anti-tuberculosis treatment was effective, and similar result was observed in our study. This also shows that 18F-FDG PET/CT has great value in detecting the efficacy of tuberculosis treatment.

We discovered a SUVmax difference between the recurrence and non-recurrence groups, so we sought to identify a discrimination cutoff point to help predict tuberculosis recurrence. The cut-off value for SUVmax was 2.15 and the area under the curve was 0.980 (95% CI 0.958 to 1.000). At this point, the sensitivity, specificity, positive predictive value and negative predictive value of PET/CT for predicting tuberculosis recurrence were 90.5%, 97.2%, 76.0% and 99.1%, respectively. The negative predictive value of 18F-FDG PET/CT imaging is very high, which means 18F-FDG PET/CT is a reliable method for eliminating the recurrence of OHPTB, which can greatly reduce the monitoring range and improve the detection efficiency. The data also indicate that the positive predictive value of 18F-FDG PET/CT is relatively low. Inflammation can increase the 18F-FDG uptake, which does not always mean an active MTB infection. In OHPTB lesions containing MTB, the immune system is able to inhibit the replication of the bacilli and prevent the recurrence of disease. 18F-FDG avidity after treatment might increase due to persistent antigens, very high-intensity or new lesion is unlikely present^18^. Malignant tumors and anti-tumor treatments will lead to immunosuppression in patients, thus disrupting the balance between the bacteria and the immune system, leading to the recurrence of OHPTB. However, the degree of inhibition varies from person to person, and predicting tuberculosis recurrence should consider the immune status of the patients.

Some scholars have used 18F-FDG-PET/CT to identify the reactivation risk in cynomolgus macaques with latent tuberculosis^1^, and another study speculated that increased 18F-FDG uptake in a OHPTB lesion might be a predictor of tuberculosis recurrence^22^, but none of these concepts had been clinically validated. In this study, we agreed that tumor and antitumor therapy cause a rapid decline in human immunity in a short time to clinically confirm the conjectures made in other studies and discovered reliable PET/CT signs that can predict the recurrence of OHPTB. However, as a retrospective study predicting the recurrence of tuberculosis in cancer patients, there were many shortcomings in the design of the study. First, many factors in this study design were uncontrollable, such as the timing of the unified image review, the degree of damage to the patients’ immunity and the duration of treatment. The follow-up time of PET/CT or CT in this study was based on the needs for tumor diagnosis and treatment, which is in line with the actual clinical status of patients. But this also led to the high rate of lost for follow-up, Although we found that there was no significant difference in demographic information (gender, age) between the lost and the final subjects (Supplementary Table), more cases are still needed to verify the results. Second, the diagnosis of tuberculosis recurrence was based on clinical and imaging follow-up, and ethical and practical limitations prevented pathological examinations from being performed in many cases, making it impossible to identify a few cases of new-onset malignant tumors or infections in the lungs. The third limitation is that we are a cancer hospital and do not have the qualifications for treating tuberculosis patients, so we can not obtain accurate microbiological data. Although we confirmed the presence of M. tuberculosis by asking the patient’s medical history, it is still impossible to completely rule out the possibility of secondary infections. Finally, the number of PET-positive cases was relatively small, making selection bias unavoidable.

## Conclusions

The high incidence and long latency of pulmonary tuberculosis make the detection of recurrence in OHPTB a long-term and difficult process. Therefore, it is very important to identify cases at high risk of recurrence to narrow the monitoring scope in the management of OHPTB patients. With SUVmax = 2.15 as the cut-off value, 18F-FDG PET/CT has high accuracy in predicting tuberculosis recurrence. Lesions with high SUVmax values have a higher risk of recurrence. Although 18F-FDG is a nonspecific tracer that may cause a false positive diagnosis, negative development is highly reliable in excluding tuberculosis recurrence. Therefore, 18F-FDG PET/CT imaging of tuberculosis is takes advantage over the other methods, providing the microbiological, immunological and physiological details in living hosts to enable early prediction of the risk of tuberculosis recurrence and improve patients’ medical decision-making.

## Materials and Methods

### Patients

This study reviewed the cancer inpatients who underwent PET/CT examinations from 2010 to 2017. The inclusion criteria for the study were as follows: 1. the patients were newly diagnosed and had not previously received antitumor therapy; 2. the presence of OHPTB was defined based on the criteria proposed by Linh *et al*.^23^ and the Centers for Disease Control and Prevention guidelines^24^; 3. the patients had detailed clinical and imaging follow-up data; and 4. follow-up lasted for more than 1 year. In total, 964 tumor patients with radiographic lesions suggesting old healed pulmonary tuberculosis were screened; 726 patients were lost to follow-up, and 238 patients were included in the final study. Of those 238 included patients, there were 77 women and 161 men, with an age range of 32–83 years (mean age, 52.3 ± 12.2 years). The patients had long histories of illness, which meant that we could only depend on oral information to obtain the approximate onset and treatment time. The average clinical cure time of the 238 patients was 23.7 years (4–56 years). This study was approved by the Shandong Cancer Hospital and Institute Affiliated with the Shandong University Center Ethics Committee and Institutional Review Boards for clinical investigations. All of the methods were performed in accordance with the Declaration of Helsinki and the relevant guidelines. All of the patients signed written informed consent forms before inclusion in the study.

### Scanning technique

Patients were imaged with a PET/CT scanner (TF Big Bore, Philips, Holland). 18F-FDG with a pH value of 5–7 and a radiochemical purity of more than 95% was produced using a cyclotron (MINItrace, GE Healthcare, Milwaukee WI, USA). The patients fasted for at least 6 h and had blood glucose levels below 200 mg/dL prior to the injection of the 18F-FDG. Patients reclined in a quiet room for 60 min after the intravenous injection of 4.4–5.5 MBq/kg of 18F-FDG.

Spiral CT scanning was performed at 120 kVp and 300 mAs, and images were reconstructed as contiguous 5 mm slices. Additional lung reformats were generated with contiguous 1 mm slices. PET was performed after spiral CT, without patient repositioning. PET images were obtained at seven to eight couch positions per patient, with an acquisition time of 1 minute in each position. We used CT scan data for attenuation correction of the PET images and fused the attenuation-corrected PET and CT images.

### Imaging analysis

The confirmation of OHPTB was based on thin slice CT; the diagnostic criteria met the criteria proposed by Linh *et al*. and the Centers for Disease Control and Prevention guidelines. FDG uptake was measured as the standardized uptake value (SUV) on the PET/CT images, and the maximum pixel value in the ROI was chosen as the maximum SUV (SUVmax).

The SUV of PET is a semiquantitative indicator with many influencing factors, so this study used more rigorous morphological criteria to confirm tuberculosis recurrence. Based on the thin slice CT images of the first PET/CT scan of the tumor patient, the recurrence criteria were confirmed as follows: (1) the old tuberculosis lesions increased in size; (2) there were new holes in the old tuberculosis lesion; or (3) there were new tuberculosis lesions around the old tuberculosis lesion. During the follow-up, PET/CT or CT scans showed that the above signs indicated tuberculosis recurrence. Indirect signs of tuberculosis recurrence (such as lymph nodes or distal organ involvement) are difficult to distinguish from those of tumor metastasis, so they are not used as criteria for evaluating tuberculosis recurrence. If the morphology of the old tuberculosis lesions had not changed for more than one year, it was determined that there was no recurrence of tuberculosis. Unfortunately, we are a cancer hospital and do not have the qualifications for treating tuberculosis patients. Due to the protection of patient data and related ethical requirements, we often only obtain the patient’s oral data and simple diagnostic reports, but can not obtain accurate data. Therefore, the patient’s microbiological data was not discussed in this study, only as a validation of our imaging diagnosis.

### Statistical analysis

All statistical analyses were performed with SPSS version 20.0. The normal distribution of continuous variables was evaluated with the Kolmogorov-Smirnov test. Comparisons of the SUVmax values between the two groups (recurrence group vs nonrecurrence group) were conducted with Student’s t test for normally distributed variables or a nonparametric test (Mann-Whitney U test) for skewed variables, which are expressed as medians and ranges. P < 0.05 was considered significant. The receiver operating characteristic (ROC) curve and the respective area under the curve (AUC) were calculated for the SUVmax. The best cut-off value was determined using the Youden index.

## Data Availability

The datasets generated and/or analyzed in the current study are available from the corresponding author on reasonable request.
